# Collodion in Mammary Inflammation and Small Pox

**Published:** 1851-03

**Authors:** J. H. Murphy

**Affiliations:** St. Anthony’s Falls, Minnesota


### ARTICLE V.

*Collodion in Mammary Inflammation and Small Pox.* By Dr. J. H. MURPHY, of St. Anthony's Falls, Minnesota.

During the month of June last, I was called upon to see a lady, afflicted with inflammation of the mamma, supervening upon confinement. It was much swollen and painful. I applied the various remedial agents usually exhibited in such cases, but with very indifferent success. I observed, in the North-Western Medical and Surgical Journal, an article, written by Prof. Evans, detailing his treatment of similar cases, which resulted favorably and speedily, by the application of the collodion. I immediately applied the remedy, in the manner designated in the paper, and, although there appeared to be but little probability of preventing suppuration, yet by the assiduous application three or four times per diem, for three days, a perfect resolution of the inflammation and dispersion of the tumor was effected, with but little or no auxiliary treatment. The success here induced me to try it in the following cases : Having at this time a case of small pox under treatment, I concluded that as it was considered an object to protect the affected surface from the action of the atmosphere, the collodion might possibly answer that purpose as well as any thing which I had at my command. As this was an experiment, however, I proceeded cautiously, applying the solution on one of the lower extremities, previous to the perfect filling of the pustules, four times daily for four successive days. From the time of the first application the pustules retrograded, the itching and irritation in the part subjected to this treatment was allayed, and after the recovery of the patient, it presented a uniform, smooth surface, while the contiguous skin, to which collodion had not been applied, was deeply pitted.

Shortly after this I had another patient, laboring under the same disease, and being greatly encouraged at the result in the previous case, concluded to make a more extensive application of the remedy. At the same period in the course of the disease, as previously mentioned, I brushed the surface of the face, neck and hands of the patient, thoroughly, three or four times daily, for four days. The result was, as in the former case, a perfect prevention of the disfiguring effects of the disease, although both patients had the affection in a severe, confluent form. In the latter case, also, the remedy succeeded most admirably in alleviating the irritation and consequent unpleasant results, usually attending the progress of the disease. Not having notes of the cases, I cannot enter into a minute description of the appearances presented at different periods, and, therefore, only give the mode and time of the application, and the result. The only unpleasant circumstance in the treatment was, that the first (small pox) patient appeared extremely chagrined that his *face* had not been subjected to the experiment instead of his *leg*, and hinted, ominously, *mal*-practice. From the experience attained in these cases, I am inclined to think collodion the most effectual means of preventing pitting in the disease referred to. It only remains to be tested by future experiment, as to the extent to which it can be applied without producing ill consequences.

As no bad results followed the application to the face and hands, in the above case, this, in itself, is a great consideration if it should be found to act as favorably in regard to the marks of the pustules hereafter. The collodion has the advantages over all other agents hitherto employed for the purpose mentioned, that it is cheaper, more easily applied, and adapts itself closely to the the inequalities of the surface, besides being so eminently useful in allaying the itching and irritation.

